# Supplementary material for: The causal relationship between the human gut microbiota and pyogenic arthritis: a Mendelian randomization study
Source: Front Cell Infect Microbiol. 2024 Nov 26;14:1452480. doi: 10.3389/fcimb.2024.1452480 (PMC11629706; doi:10.3389/fcimb.2024.1452480)
Supplement: Supplementary file 1 [file DataSheet1.zip › mendelian test/ebi-a-GCST90027542.csv_leave_one_out.pdf]

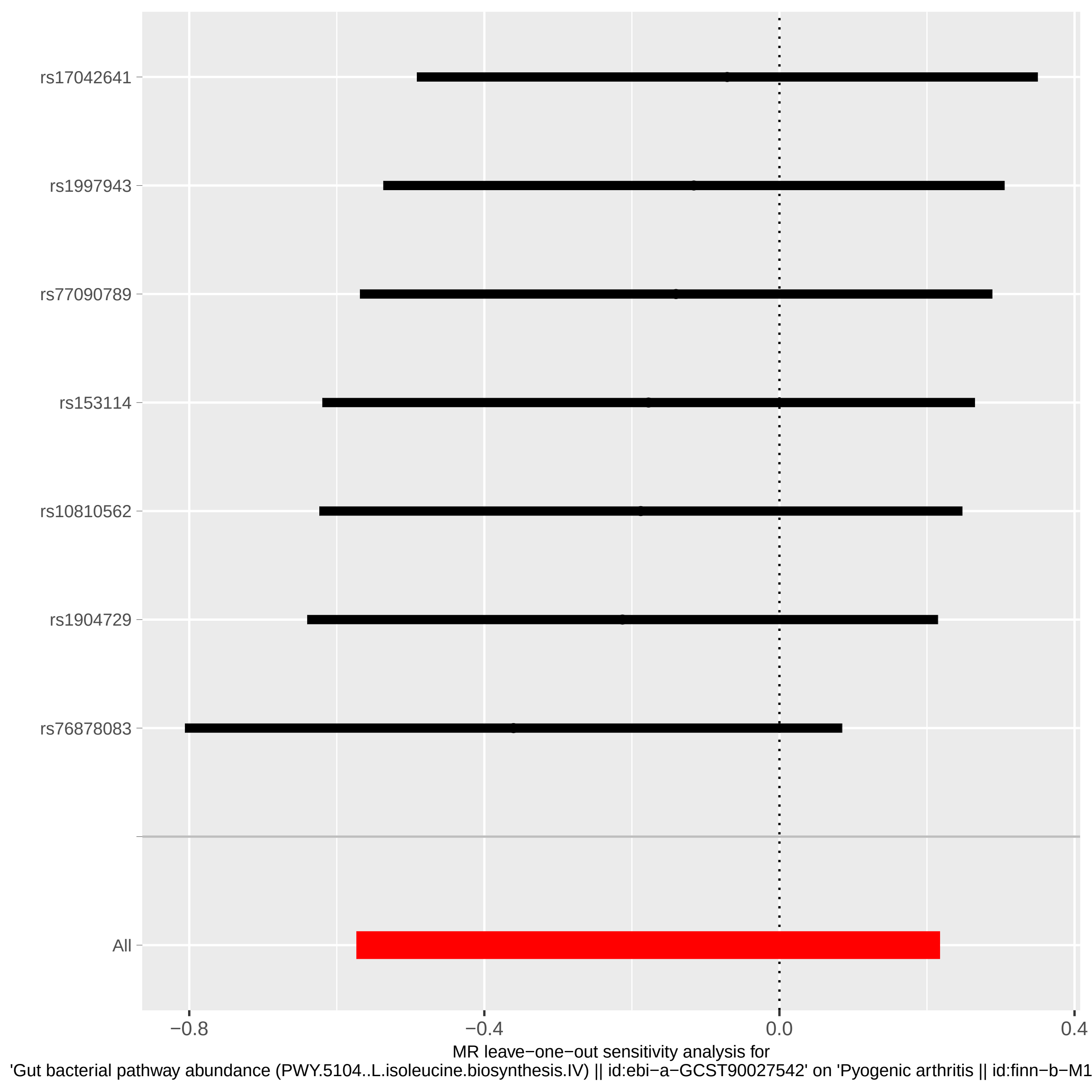
rs17042641
rs1997943
rs77090789
rs153114
rs10810562
rs1904729
rs76878083
All
−0.8
−0.4
0.0
0.4
MR leave−one−out sensitivity analysis for
'Gut bacterial pathway abundance (PWY.5104..L.isoleucine.biosynthesis.IV) || id:ebi−a−GCST90027542' on 'Pyogenic arthritis || id:finn−b−M1
